# Actitudes y comportamientos relacionados con salud bucal según la experiencia de caries en jóvenes aspirantes a soldados paraguayos. Un estudio transversal

**DOI:** 10.21142/2523-2754-1301-2025-226

**Published:** 2025-03-03

**Authors:** Clarisse Díaz-Reissner, Diego Ávalos González, Maria Silvana Carabajal, Maria Elida Quintana-Molinas, Milner I. Morel-Barrios

**Affiliations:** 1 Universidad Nacional de Asunción, Facultad de Odontología. Asunción, Paraguay. cdiazr@founa.edu.py diegoavalos@founa.edu.py msilvanacarabajal@founa.edu.py mariae.odontomeqm@gmail.com milnermorel@founa.edu.py Universidad Nacional de Asunción Universidad Nacional de Asunción Facultad de Odontología Asunción Paraguay cdiazr@founa.edu.py diegoavalos@founa.edu.py msilvanacarabajal@founa.edu.py mariae.odontomeqm@gmail.com milnermorel@founa.edu.py

**Keywords:** salud bucal, actitud frente a la salud, conducta, personal militar (DeCS/BIREME), oral health, attitudes towards health, behavior, military personnel (MeSH/NLM)

## Abstract

**Objetivo:**

Asociar las actitudes y comportamientos relacionados con la salud bucal con la experiencia de caries en aspirantes a soldados de la Brigada Aerotransportada de la Fuerza Aérea Paraguaya en 2023.

**Materiales y métodos:**

El diseño fue transversal. La muestra estuvo comprendida por 100 soldados. Se aplicó el Inventario de Conducta Dental de la Universidad de Hiroshima (HU-DBI) para medir las actitudes y comportamientos relacionados con la salud bucal, y evaluar la experiencia de caries mediante el índice CPO-D. Se aplicó la prueba chi cuadrado de Pearson para comprar la distribución del HU-DBI y CPO-D según procedencia, y el test exacto de Fisher para asociar la experiencia de caries con las actitudes y comportamientos con el nivel de confianza del 95%.

**Resultados:**

En cuanto a la valoración de actitudes y comportamientos, la puntuación promedio fue de 6,33 (DE = 1,97), considerado como “regular”, y la puntuación mínima obtenida fue 2 y la máxima fue 11. El índice CPO-D fue 5,45 (DE = 3,9). Se encontró una distribución homogénea entre las actitudes y comportamientos (p = 0,197), tampoco la experiencia de caries con la procedencia (p = 0615). No se encontró asociación entre la experiencia de caries con las actitudes y comportamientos (p = 0,577).

**Conclusión:**

Las actitudes y comportamientos sobre salud bucal fue regular, la experiencia de caries dental obtenidos por el índice CPO-D fue baja. No se encontró asociación entre ambas variables.

## Abstract (en)

**Objective:**

To associate attitudes and behaviors related to oral health with the experience of dental caries in trainees of the Paraguayan Air Force's Airborne Brigade in the year 2023.

**Materials and methods:**

The design was cross-sectional. The sample comprised 100 soldiers. The Hiroshima University Dental Behavior Inventory (HU-DBI) was applied to measure attitudes and behaviors related to oral health, and the experience of caries was assessed using the DMFT index. The Pearson Chi-square test was used to compare the distribution of HU-DBI and DMFT according to background, and Fisher's exact test was applied to associate the experience of caries with attitudes and behaviors at a 95% confidence level.

**Results:**

: Regarding the assessment of attitudes and behaviors, the average score was 6.33 (SD=1.97), classified as "fair," with a minimum score of 2 and a maximum of 11. The DMFT index was 5.45 (SD=3.9). A homogeneous distribution was found between attitudes and behaviors (p=0.197), and there was no association between caries experience and background (p=0.615). No association was found between caries experience and attitudes and behaviors (p=0.577).

**Conclusion:**

The attitudes and behaviors regarding oral health were fair, and the experience of dental caries measured by the DMFT index was low. No association was found between the two variables.

## INTRODUCCIÓN

La carga global de los problemas de salud bucodental está aumentando en todo el mundo, en países de ingresos bajos y medianos, es probable que la carga general de demanda de dichos servicios siga aumentando debido al crecimiento y el envejecimiento progresivo de la población ^1^. En Latinoamérica, en la mayoría de los países, la salud bucal no es un tema de salud prioritario ^2^. Las enfermedades orales más comunes son la caries dental, la periodontitis grave, la pérdida de dientes y el cáncer oral ^3^. 

Para evaluar las actitudes y comportamientos relacionado con salud bucal se ha desarrollado el Inventario de Conducta Dental de la Universidad de Hiroshima, conocido por su siglas en inglés HU-DBI (The Hiroshima University Dental Behaviour Inventory) que fue creado por Kawamura ^4^. Dicho cuestionario ha demostrado ser útil para la evaluación de las actitudes y el comportamiento de salud bucal, y ha sido efectivamente utilizado para comparaciones transculturales, el cuestionario se ha traducido con éxito a varios idiomas ^5^. 

Durante la Primera Guerra Mundial, la deficiente higiene dental entre los soldados puso de manifiesto que la boca en las trincheras era un problema de salud importante, agravado por la falta de atención dental y las adversas condiciones de vida en las trincheras ^6^. A pesar de los avances en la atención dental, los problemas persisten, ya que los estudios indican tasas elevadas de problemas dentales entre el personal militar, lo que subraya la necesidad de mejorar la atención dental y las estrategias preventivas ^7^. El personal militar se enfrenta con frecuencia a niveles elevados de estrés, lo que repercute negativamente en las prácticas de higiene bucal, ya que el estrés se correlaciona con el descuido de las rutinas de cuidado personal. Esto aumenta el riesgo de enfermedad periodontal, mientras que la dieta militar puede ser deficiente en nutrientes vitales necesarios para mantener la salud bucal, lo que agrava la incidencia de enfermedades dentales entre los soldados ^8^.

En Paraguay, los datos epidemiológicos sobre salud bucodental y utilización de servicios en adultos son limitados, por lo que parece una necesidad generar información para diseñar e implementar programas que atiendan las necesidades de esta población. En Paraguay, el 98% de la población sufre de problemas bucodentales. La prevalencia de caries de la población general es del 63,31%; la mayor prevalencia se presentó en el área rural (74,58%), mientras que en el área urbana fue del 57,75%. Este comportamiento fue el mismo para todas las edades ^9^. En este estudio se decidió aplicar un inventario de conductas dentales para investigar las actitudes y comportamientos de los aspirantes, así como para conocer sus costumbres de cuidado bucal, dado que en Paraguay no existen antecedentes de su aplicación. Esto resulta importante porque la salud bucal es fundamental para la capacidad de los soldados para cumplir con sus deberes y responsabilidades. Los problemas de origen dental pueden causar dolor, inflamación y otros síntomas que afectan la capacidad del soldado para realizar sus tareas diarias. Además, la falta de atención dental adecuada puede llevar a problemas más graves en el futuro, como la pérdida de dientes y la enfermedad periodontal. Por esto se planteó como objetivo asociar las actitudes y comportamientos relacionados con la salud bucal con la experiencia de caries en los aspirantes a soldados profesionales de la Brigada Aerotransportada de la Fuerza Aérea Paraguaya de la ciudad de Luque, en 2023.

## MATERIALES Y MÉTODOS

El diseño del estudio fue transversal. La muestra estuvo compuesta por un total de 100 aspirantes a soldados profesionales. Se incluyó a mayores de 18 años. Se excluyó a aquellos que estaban indispuestos o aislados por motivos de salud, quienes estaban realizando actividades y los que no aceptaron participar. El protocolo de investigación cumplió con los principios éticos y contó con Aprobación del Comité de Ética en Investigación de la Facultad de Odontología de la Universidad Nacional de Asunción (Informe N.° 017/2023), así como no generó ningún riesgo para los encuestados. Todos los aspirantes a soldados fueron invitados a participar libre y voluntariamente, previa firma del consentimiento informado. La encuesta no recopiló datos sensibles de los participantes, por lo que se garantizó la confidencialidad. Se utilizaron las fichas del archivo del consultorio odontológico de la Sanidad de la Brigada Aerotransportada de la Fuerza Aérea Paraguaya para medir el índice de CPO-D. A fin de valorar la práctica en salud bucal se aplicó el inventario de HU-DBI que consta de 20 preguntas, de 1 a 8 corresponde a actitudes sobre salud bucal, de 9 a 16 corresponde a comportamientos sobre salud bucal y de 17 a 20 corresponde a autovaloración sobre salud bucal. Dicho inventario valora la práctica de salud bucal de las personas, y la máxima puntuación de actitudes y comportamientos es 12. Se dio como resultado “bueno” si el valor se encuentra entre 8 y 12, “regular” cuando es entre 4 y 7 y “malo” cuando es de 0 a 3 ^10^. Para realizar la puntuación se valoraron las siguientes preguntas de la encuesta: 5, 7, 8, 9, 11, 12, 13 y 14. Si estas fueron marcadas “Sí”, se le asigna un punto a cada una, mientras que en las preguntas 2, 4, 18 y 19 se debe asignar un punto si fueron marcadas “No”. La suma de todos los puntos obtenidos, previamente valorados por pregunta, da como resultado el índice HU-DBI, que tiene cada persona encuestada ^11^. Los datos obtenidos fueron tabulados en Microsoft Office Excel 365. Se aplicó la prueba de chi cuadrado de Pearson para comparar la distribución de las variables inventario HU-DBI e índice CPO-D con la procedencia, y el test exacto de Fisher para evaluar la asociación entre la experiencia de caries con las actitudes y comportamientos, con un nivel de confianza del 95%. Se utilizó el programa Epi Info® 7.

## RESULTADOS

Participaron 100 aspirantes a soldados profesionales de la Brigada Aerotransportada de la Fuerza Aérea Paraguaya en 2023. El rango etario que predominó fue de 18 a 20 años, lo que corresponde al 82%. El departamento de procedencia con mayor cantidad de encuestados fue central, con un porcentaje del 45% (Tabla 1).

**Tabla 1:** Distribución demográfica (N = 100)

Variables	Categoría	Porcentaje
Edad (años)	18-20	82%
	21-25	13%
	26-29	4%
Procedencia	Capital - Asunción	45%
	Interior del país	55%

Las preguntas respondidas de forma casi unánime de manera positiva por los soldados fueron: “si se preocupa por el color de sus dientes” (92%) y “si se preocupa cuando tiene mal aliento” (96%). Por otro lado, una de las preguntas con frecuencias similares en ambas categorías fue “si ha notado algunos depósitos pegajosos (sarro) en sus dientes”, el 51% respondió “Sí”, el 49% respondió “No” (Tabla 2).

**Tabla 2:** Respuestas a la prueba HU-DBI

Preguntas	Sí	No
1. ¿Se preocupa por el color de sus dientes?	92%	8%
2. ¿Cree que usaría prótesis dental en un futuro?	23%	77%
3. ¿Está preocupado por el color de sus encías?	78%	22%
4. ¿Cree que sus dientes están empeorando a pesar del cepillado diario?	32%	68%
5. ¿Cree que puede limpiarse bien los dientes sin usar crema dental?	42%	58%
6. ¿Se preocupa cuando tiene mal aliento?	96%	4%
7. ¿Cree prevenir la enfermedad de las encías solo con cepillar sus dientes?	38%	62%
8. ¿Usted toma mucho tiempo para cepillarse los dientes?	16%	84%
9. ¿Ha notado algunos depósitos pegajosos (sarro) en sus dientes?	51%	49%
10. ¿Usa un cepillo de dientes de cabeza pequeña?	49%	51%
11. ¿Le han enseñado profesionalmente a cepillarse los dientes?	87%	13%
12. ¿Se cepilla cada uno de los dientes con cuidado?	35%	65%
13. ¿A menudo revisa sus dientes después de cepillarse?	60%	40%
14. ¿Usa hilo dental a diario?	22%	78%
15. ¿Utiliza cepillo de dientes de cerdas duras?	44%	56%
16. ¿Siente limpios sus dientes solo si lo cepilla enérgicamente?	57%	43%
17. ¿Se preocupa de ir regularmente al odontólogo?	64%	36%
18. ¿Sus encías sangran al cepillarse los dientes?	43%	57%
19. ¿Solo el dolor de dientes es lo que le hace ir al odontólogo?	51%	49%
20. ¿Ha oído a su odontólogo decir que se cepilla bien los dientes?	45%	55%

**Figura 1:**
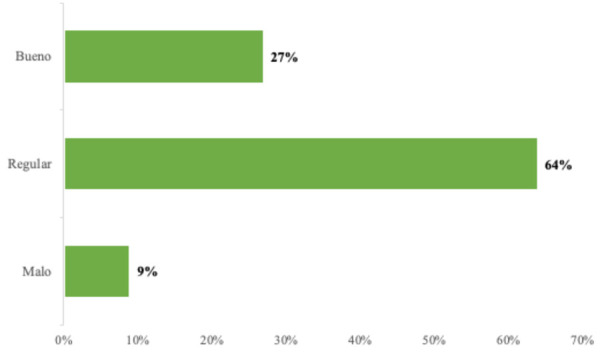
Actitudes y comportamientos relacionados con la salud bucal según el índice HU-DBI

Del total de soldados encuestados, el 64% obtuvo un índice “regular”, que fue el mayor porcentaje (Figura 1).

El puntaje promedio del inventario HU-DBI fue de 6,33 (DE = 1,97), considerado como “regular”, y se obtuvo una puntuación mínima de 2 y una máxima de 11.

El resultado del índice CPO-D de la muestra de la población fue de un valor promedio de 5,45, que se clasifica como “bajo” dentro de los criterios de valoración, con una desviación estándar de 3,9 (Tabla 4).

**Tabla 4:** Medias de tendencia central y dispersión del Índice CPO-D

Dientes	Media	Desviación estándar
Cariados	2,24	3,2
Perdidos	1,97	2,3
Obturados	1,21	2,1
CPO-D	5,45	3,9

La distribución fue homogénea entre el inventario HU-DBI y la procedencia de los participantes, así como el índice CPO-D y la procedencia (Tabla 5).

**Tabla 5:** Inventario HU-DBI e índice CPO-D por procedencia

Variables	Categorías	Urbana	Rural	Total	p-valor
Inventario HU-DBI	Bueno	15	12	27	0,197
	Malo/Regular	30	43	73	
	Total	45	55	100	
Índice CPO-D	Moderado/Alto	10	10	20	0,615
	Bajo/Muy bajo	35	45	80	
	Total	45	55	100	

No se encontró asociación entre la experiencia de caries con las actitudes y comportamientos (Tabla 6).

**Tabla 6:** Asociación entre experiencia de caries con las actitudes y comportamiento

Índice CPO-D	HU-DBI		
	Malo/Regular	Bueno	Total
Muy bajo/Bajo	57	23	80
Moderado/Alto	16	4	20
Total	73	27	100

p = 0,577

## DISCUSIÓN

En la presente investigación se evaluaron las actitudes y comportamientos, y se obtuvo una puntuación promedio de 6,33 del inventario HU-DBI, resultado similar al reportado en una población de estudiantes de odontología árabes en el cual la puntuación media del inventario fue de 6,31, que fue también regular ^12^.

En una población de soldados de 18 a 29 años, el 79% presentó buenas actitudes, resultado similar al de la presente investigación, en donde la mayoría de los encuestados (63%) presentaron buenas actitudes ^13^. En otro estudio compararon una población de estudiantes de odontología del último año procedentes de Finlandia y Japón, y los conocimientos sobre salud bucal resultaron ser muy diferentes entre ellos. Esto reflejaba una diferencia entre la cultura o el sistema de educación de salud en ambos países: en los resultados de dicha investigación solo el 2% de los estudiantes finlandeses posponían una cita al odontólogo hasta sentir dolor, mientras que el 56% de los estudiantes japoneses manifestaban lo mismo. En la presente investigación se obtuvo un 51%, cifra similar a la de Japón. Solo el 7% de los estudiantes finlandeses respondió “creo que mis dientes están empeorando a pesar del cepillado diario”, mientras que un 30% de los estudiantes japonés respondió lo mismo ^14^. Al realizar una comparación con estudios realizados en distintos países se pudo apreciar que, al momento de evaluar las actitudes y comportamientos relacionados a salud bucal en distintas poblaciones, el impacto cultural presenta un gran peso.

Se estableció una asociación entre las actitudes y los comportamientos relacionados con la salud bucal y la procedencia de los soldados participantes, y se determinó que no existe asociación entre ambas variables. En un mundo cada vez más globalizado, las barreras culturales y geográficas se desvanecen, ya que personas de diferentes procedencias se unen en diversas esferas de la vida, incluyendo el ámbito militar. En este contexto, es importante reconocer que la procedencia de los soldados no debe ser un factor determinante para evaluar sus actitudes y conocimientos sobre salud bucal, ya que los mismos se basan en la educación, la experiencia, el acceso a información actualizada y la conciencia individual sobre la importancia de mantener una buena higiene oral.

Los resultados del índice CPOD de 5,45 interpretados como bajo coinciden con los resultados de la investigación realizada en nuestro país en una población de adultos de 18 a 29 años, donde el 50,4% de la población examinada presentó un CPO-D bajo ^15^.

Por otro lado, no existió una asociación entre el índice CPO-D y la procedencia de los participantes. Si bien, la procedencia de los participantes está relacionada a factores como la accesibilidad a servicios prestadores de salud.

La caries dental es el resultado de una combinación individual de factores en la vida de cada individuo y es importante reconocer que, si bien las actitudes y comportamientos sobre salud bucal pueden influir en cierta medida, son prácticas importantes para mantener una buena salud, por ejemplo, saber cómo cepillarse adecuadamente, cómo usar hilo dental, pero no son los únicos determinantes. Al conversar con los participantes, gran parte de ellos manifestaron que no pueden cepillarse adecuadamente o usar hilo dental debido al limitado tiempo que presentan entre sus actividades de preparación militar.

El entrenamiento físico y táctico en el campo es una parte fundamental de la formación de los soldados. Esta profesión es conocida por la disciplina que exige, por lo que consideramos fundamental abordar la salud de los reclutas de manera integral, incluyendo las afecciones orales en esta evaluación. Aunque no forma parte del presente estudio, sería valioso que investigaciones futuras incluyan un análisis de la situación socioeconómica de los participantes, ya que el nivel socioeconómico forma parte de los múltiples determinantes sociales del estado de salud.

### Limitaciones

Se puede mencionar que no todas las preguntas realizadas en el inventario HU-DBI en idioma español, eran entendidas por algunos aspirantes, motivo por el cual ciertas preguntas fueron traducidas al idioma guaraní por los investigadores al momento de recabar las respuestas.

## CONCLUSIONES

Las actitudes y comportamientos sobre la salud bucal resultaron regulares en la mayoría de los aspirantes a soldados profesionales de la Brigada Aerotransportada de la Fuerza Aérea Paraguaya. En cuanto al resultado de experiencia de caries dental obtenido por el índice CPO-D, este fue bajo y no se encontró asociación entre el inventario HU-DBI y el índice CPO-D.
